# The Ccr4-Not Complex Interacts with the mRNA Export Machinery

**DOI:** 10.1371/journal.pone.0018302

**Published:** 2011-03-28

**Authors:** Shana C. Kerr, Nowel Azzouz, Stephen M. Fuchs, Martine A. Collart, Brian D. Strahl, Anita H. Corbett, R. Nicholas Laribee

**Affiliations:** 1 Department of Biochemistry, Emory University School of Medicine, Atlanta, Georgia, United States of America; 2 Department of Biochemistry, Cell, and Developmental Biology Graduate Program, Emory University School of Medicine, Atlanta, Georgia, United States of America; 3 Department of Microbiology and Molecular Medicine, University of Geneva Medical School, Geneva, Switzerland; 4 Department of Biochemistry and Biophysics, University of North Carolina School of Medicine, Chapel Hill, North Carolina, United States of America; 5 Department of Pathology and Laboratory Medicine and Center for Cancer Research, University of Tennessee Health Sciences Center, Memphis, Tennessee, United States of America; Beckman Research Institute of the City of Hope, United States of America

## Abstract

**Background:**

The Ccr4-Not complex is a key eukaryotic regulator of gene transcription and cytoplasmic mRNA degradation. Whether this complex also affects aspects of post-transcriptional gene regulation, such as mRNA export, remains largely unexplored. Human Caf1 (hCaf1), a Ccr4-Not complex member, interacts with and regulates the arginine methyltransferase PRMT1, whose targets include RNA binding proteins involved in mRNA export. However, the functional significance of this regulation is poorly understood.

**Methodology/Principal Findings:**

Here we demonstrate using co-immunoprecipitation approaches that Ccr4-Not subunits interact with Hmt1, the budding yeast ortholog of PRMT1. Furthermore, using genetic and biochemical approaches, we demonstrate that Ccr4-Not physically and functionally interacts with the heterogenous nuclear ribonucleoproteins (hnRNPs) Nab2 and Hrp1, and that the physical association depends on Hmt1 methyltransferase activity. Using mass spectrometry, co-immunoprecipitation and genetic approaches, we also uncover physical and functional interactions between Ccr4-Not subunits and components of the nuclear pore complex (NPC) and we provide evidence that these interactions impact mRNA export.

**Conclusions/Significance:**

Taken together, our findings suggest that Ccr4-Not has previously unrealized functional connections to the mRNA processing/export pathway that are likely important for its role in gene expression. These results shed further insight into the biological functions of Ccr4-Not and suggest that this complex is involved in all aspects of mRNA biogenesis, from the regulation of transcription to mRNA export and turnover.

## Introduction

Gene expression is regulated at multiple levels, including at the stages of transcriptional and post-transcriptional control, to achieve correct levels and patterns of expression [1]. The nuclear steps required for gene expression are highly integrated and are controlled by evolutionarily conserved factors and mechanisms which package an mRNA molecule into an export-competent ribonucleoprotein (mRNP) complex [1], [2], [3]. There is mounting evidence that the steps from transcription to mRNA export are not only sequential, but in fact are highly coupled and interdependent, whereby proteins involved in one step of mRNA biogenesis are subsequently used as adaptor proteins to recruit other processing or export factors [1], [2], [3], [4], [5], [6]. Among these RNA binding proteins are the historically defined heterogenous nuclear ribonucleoproteins (hnRNPs) which mediate multiple steps in the mRNA lifecycle such as processing, nuclear export, and delivery to the cytoplasm [7], [8]. The budding yeast *Saccharomyces cerevisiae* has a number of hnRNPs including Hrp1, which is required for proper mRNA cleavage and polyadenylation [9], the poly(A) binding protein, Nab2, required for mRNA export and proper poly(A) tail length [10], [11], [12], and Npl3, which is involved in splicing, transcription elongation, and export [13], [14].

Following mRNA maturation and processing, the export-competent mRNP must travel through the nuclear pore complex (NPC) to reach the cytoplasm. The NPC is an evolutionarily conserved structure comprised of approximately 30 protein components called nucleoporins (Nups), which are present in at least 8 copies per NPC and are arranged in 8-fold radial symmetry to form channels that perforate the nuclear envelope and mediate traffic between the nucleus and cytoplasm [15], [16]. Some Nups are asymmetrically localized across the NPC, giving the complex three distinct substructures: a nuclear basket, a central core spanning the nuclear envelope, and cytoplasmic fibrils [16]. In order for an mRNA to translocate through the NPC, mRNA export factors in complex with the mRNA interface with a distinct class of Nups called FG-Nups, which contain at least one domain of distinct repeating patterns of phenylalanine (F) and glycine (G) residues [5], [16]. Mutations in many distinct Nups result in mRNA export defects and mRNA accumulation in the nucleus [17], [18], [19], [20], [21], [22], [23]. Interestingly, recent studies have uncovered a physical link between transcriptionally active genes and the NPC [24], reminiscent of Blobel's gene gating hypothesis [25] and further suggesting that every aspect of mRNA maturation may be tightly coupled from biogenesis to nuclear export.

A significant contributor to the lifecycle of an mRNA molecule, from mRNA biogenesis to eventual degradation, is the evolutionarily conserved multi-subunit Ccr4-Not complex. The Ccr4-Not complex is a large protein complex (∼0.9–1.0 MDa), containing nine core subunits (Ccr4, Caf1, Caf40, Caf130, and Not1-5) that localizes to both the nucleus and cytoplasm [26], [27]. The Caf1 and Ccr4 subunits are mRNA deadenylases, responsible for the major cytoplasmic deadenylase activity in budding yeast [28], [29], [30], The Not4 subunit is a RING-domain containing ubiquitin ligase whose only known substrates are the Egd1 and Egd2 proteins involved in translation and the Jhd2 histone demethylase [31], [32], [33]. The Ccr4-Not complex negatively and positively regulates both transcription initiation and elongation, and it has been suggested that the combined actions of Ccr4-Not members contribute to transcriptional control of ∼85% of the *S. cerevisiae* genome [34], [35]. This regulation is achieved in part through physical interactions between Ccr4-Not subunits and components of the basal transcription apparatus and other accessory transcriptional co-regulators, including the SAGA histone acetyltransferase complex, the PAF transcription elongation complex, and the proteasome [36], [37], [38], [39], [40].

Until recently, the known nuclear functions of Ccr4-Not were confined to transcriptional regulation; however, new studies suggest that Ccr4-Not contributes significantly to other nuclear processes. Cells mutant for Ccr4-Not components show an increase in the steady state levels of both snRNAs and snoRNAs and accumulate a significant fraction of these RNAs as polyadenylated species [41]. Ccr4-Not also interacts physically and functionally with both the nuclear exosome and the TRAMP complex, components of a nuclear surveillance pathway that targets aberrantly processed RNAs for degradation [41]. These results suggest that Ccr4-Not has a role in nuclear RNA turnover through interactions with both the exosome and TRAMP. Ccr4-Not also has been linked to other nuclear, RNA-based processes. For example, one of the two human Caf1 orthologs, hCaf1, associates with the arginine methyltransferase, PRMT1 [42]. Both factors localize to nuclear speckles, which are sub-nuclear domains enriched for small nuclear ribonucleoproteins and splicing factors. hCAF1 interaction with PRMT1 regulates PRMT1-mediated methylation of both histone H4 and the RNA binding protein Sam68 *in vitro* and *in viv*o, suggesting that Ccr4-Not may play a significant role in PRMT1-regulated biological processes, including mRNA processing and export.

To further define the interactions between Ccr4-Not and processes regulated by arginine methylation, we used budding yeast to determine whether Ccr4-Not members interact with the yeast ortholog of PRMT1, the hnRNP methyltransferase Hmt1 [43]. In this study, we demonstrate that Ccr4-Not subunits physically and functionally interact both with Hmt1 and the hnRNPs, Hrp1 and Nab2, which are Hmt1 substrates [44], [45]. We also identify physical and functional interactions between Ccr4-Not subunits and multiple NPC components, and implicate these interactions in mRNA export. These studies suggest a novel functional role for Ccr4-Not in the mRNA processing/export pathway that likely depends on interactions with Hmt1, hnRNPs, and the NPC.

## Results

### Hmt1 physically interacts with components of the Ccr4-Not complex

**Figure 1:**
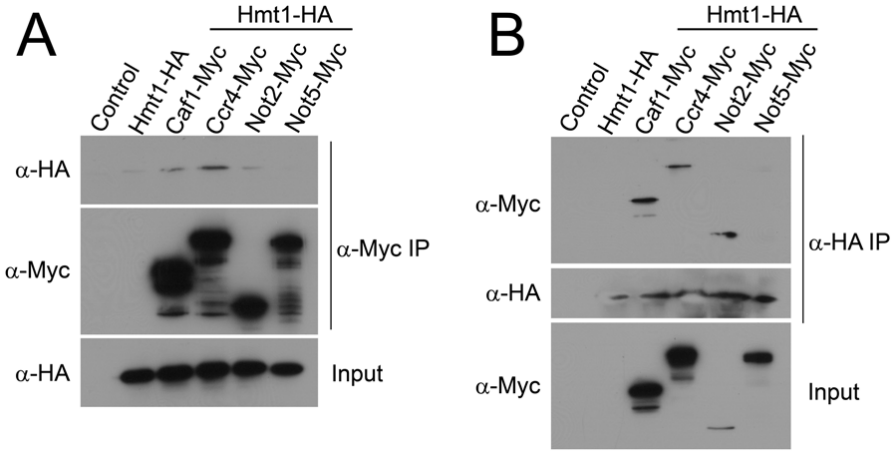
Ccr4-Not subunits associate with the arginine methyltransferase Hmt1. (A) Cells expressing the indicated Myc-tagged Ccr4-Not subunit as well as HA-tagged Hmt1 were used to examine the interaction between the Ccr4-Not complex and Hmt1. Cells were grown and lysates prepared as described in Materials and Methods. Ccr4-Not subunits were immunoprecipitated with α-Myc antibody (α-Myc IP) and the co-immunoprecipitation of Hmt1 was assessed by immunoblotting with an α-HA antibody (α-HA). The efficiency of immunoprecipitation was assessed by probing the same blots with α-Myc antibody (α-Myc). Input samples (30 µg total lysate) were probed with α-HA antibody to detect HA-tagged Hmt1 (α-HA). As controls, cells expressing no tagged protein (Control) or only Hmt1-HA show no co-immunoprecipitation. (B) The same samples in (A) were subjected to immunoprecipitation with α-HA antibody (α-HA IP) to detect co-immunoprecipitation with the Myc-tagged Ccr4-Not subunits. Immunoblots were probed with α-Myc antibody (α-Myc) to detect Ccr4-Not subunits or α-HA antibody (α-HA) to assess the level of Hmt1 precipitated. Input samples (30 µg total lysate) were probed with α-Myc to detect Ccr4-Not subunits (α-Myc). As controls, cells expressing no tagged protein (Control) or only Hmt1-HA show no co-immunoprecipitation.

A previous study identified one of the human homologs of yeast Caf1, hCAF1, as a regulator of the arginine methyltransferase PRMT1 [42]. To determine whether this functional relationship is evolutionarily conserved in the yeast *Saccharomyces cerevisiae*, we performed co-immunoprecipitation experiments to determine whether Hmt1, the budding yeast ortholog of PRMT1 [43], physically associates with components of the Ccr4-Not complex. For this analysis, an HA-tag was integrated into the endogenous *HMT1* locus in cells also expressing Myc-tagged Ccr4-Not subunits from their endogenous loci. We then performed co-immunoprecipitation experiments from whole-cell lysates with α-Myc antibody to precipitate individual Ccr4-Not subunits and blotted with α-HA antibody to detect Hmt1 association. Hmt1 association is readily detectable with the Caf1 and Ccr4 subunits, and is more weakly detected with the Not2 subunit (Figure 1A). Interestingly, Hmt1-HA did not co-immunoprecipitate with Not5-Myc, suggesting the possibility that differential interactions occur between Hmt1 and the individual Ccr4-Not members or that Not5 association is less stable than the other subunits. We confirmed these results by performing the reciprocal co-immunoprecipitations and obtained similar results (Figure 1B).

### The Ccr4-Not complex physically interacts with hnRNPs

Among the major physiological targets of the Hmt1 methyltransferase are heterogeneous nuclear ribonucleoproteins (hnRNPs) [43], [44], which bind mRNAs during processing and export from the nucleus [7], [8]. In *S. cerevisiae*, hnRNPs include Hrp1, Nab2, and Npl3 [43], [44], [45].

**Figure 2:**
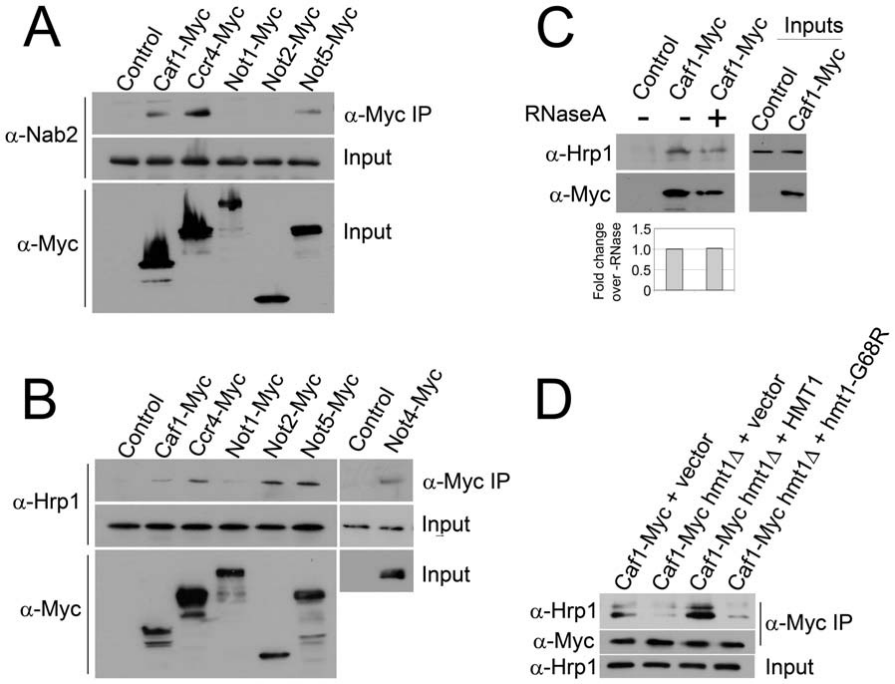
Association of the hnRNP proteins Hrp1 and Nab2 with Ccr4-Not subunits depends on Hmt1 arginine methyltransferase activity. (A) Nab2 associates with Caf1, Ccr4, and Not5. Cells expressing the indicated Myc-tagged Ccr4-Not subunit were used in co-immunoprecipitation experiments as described in the Methods to examine the interaction between the Ccr4-Not complex and Nab2. As a control, cells expressing no tagged protein (Control) show no co-immunoprecipitation. Nab2 co-immunoprecipitation and input levels (30 µg) were detected by α-Nab2 immunoblotting while Ccr4-Not subunits detected by α-Myc immunoblotting. (B) Hrp1 associates with Caf1, Ccr4, Not1, Not2 and Not5. Experiments were performed as in (A) and the co-immunoprecipitation of Hrp1 was assessed by immunoblotting with α-Hrp1 antibody (α- Hrp1). *C*, The interaction between Hrp1 and Caf1 is not RNase-sensitive. Cells expressing Caf1-Myc were grown and lysed as described in Materials and Methods. Lysate was divided into two samples and one aliquot was treated with RNase A prior to immunoprecipitation with α-Myc antibody as described in the Methods. Fold change in co-immunoprecipitation was determined using reverse image scanning densitometry as described in the Methods. *D*, Caf1 association with Hrp1 depends on Hmt1-mediated arginine methylation. *CAF1-MYC hmt1Δ* cells were transformed with empty vector, *HMT1* or *hmt1G68R* expression vectors, and cells were grown in SC-Ura media to select for plasmid maintenance. Lysates were prepared as described in Materials and Methods. Caf1-Myc was immunoprecipitated with α-Myc antibody (α-Myc IP) and the co-immunoprecipitation of Hrp1 was assessed by immunoblotting with α-Hrp1 antibody (α-Hrp1). Efficiency of immunoprecipitation was assessed by probing the same blots with α-Myc antibody. Input samples (30 µg total lysate) were probed with α-Myc and α-Hrp1 antibody. Fold change in co-immunoprecipitation was determined using reverse image scanning densitometry as described in Methods.

Given the physical association we identified between Hmt1 and Ccr4-Not subunits, we hypothesized that Ccr4-Not might also interact with hnRNPs. To investigate this possibility, we prepared lysates from cells expressing Myc-tagged Ccr4-Not subunits, performed α-Myc immunoprecipitations and then immunoblotted with antibodies specific for either Nab2 or Hrp1 to determine if they co-precipitated with Ccr4-Not members. Nab2 co-immunoprecipitates with Caf1, Ccr4, and Not5, but not with Not1 or Not2 (Figure 2A). Similar to results for Nab2, we identified Hrp1 in co-immunoprecipitates of Caf1, Ccr4, and Not5. Interestingly, in contrast to the results for Nab2 association, we did detect Hrp1 in the Not2 and Not1 immunoprecipitates (Figure 2B). As a control, Nab2 and Hrp1 did not co-immunoprecipitate from cell lysates that did not express Myc-tagged Ccr4-Not subunits. We also find that Hrp1 co-immunoprecipitates with Not4 (Figure 2B). Given the role of Ccr4-Not in transcriptional regulation, coupled with the fact that Hrp1 and Nab2 are RNA binding proteins, interactions between Ccr4-Not subunits and hnRNPs could be RNA-mediated. We therefore repeated these immunoprecipitation experiments after pretreating lysates with RNase. These experiments reveal that these interactions do not depend on the presence of RNA, as interactions between Caf1 and Hrp1 or Caf1 and Nab2 were not reduced following RNase treatment (Figure 2C and data not shown). These results suggest that interactions between the Ccr4-Not complex and hnRNPs may be mediated by either direct or indirect protein-protein interactions. Taken together, these experiments reveal that multiple subunits of the Ccr4-Not complex differentially associate with the hnRNPs Nab2 and Hrp1, and they further suggest that Ccr4-Not may play a functional role in the mRNA export pathway.

### Physical interactions between the Ccr4-Not complex and hnRNPs depend upon Hmt1 methyltransferase activity

To test whether the association between Ccr4-Not members and hnRNPs is Hmt1-dependent, we deleted *HMT1* in cells expressing Caf1-Myc and tested for association with the hnRNP, Hrp1. While we detected Hrp1 in the Caf1-Myc immunoprecipitates, the amount of Hrp1 co-immunoprecipitated with Caf1-Myc was profoundly reduced in *hmt1Δ* cells relative to cells expressing *HMT1* (Figure 2D), suggesting that the interaction between Caf1 and Hrp1 is Hmt1-dependent. Furthermore, these interactions depend upon the methyltransferase activity of Hmt1, as Caf1 does not interact significantly with Hrp1 when the catalytically-inactive *hmt1-G68R* mutant [46] is expressed in *hmt1Δ* cells, whereas expression of wildtype *HMT1* does restore these interactions. These results support the hypothesis that Ccr4-Not members associate both with Hmt1 and hnRNPs, and these associations are dependent on Hmt1 methyltransferase activity. Using an antibody specific to arginine-methylated Npl3 [47], [48], we find no significant differences in Hmt1-dependent methylation of Npl3 in Ccr4-Not deletion mutants compared to wild-type cells (data not shown), suggesting that perturbation of the Ccr4-Not complex does not impact Hmt1 methyltransferase activity *in vivo*. Taken together, these results suggest that methylation of Hrp1 and Nab2 by Hmt1 is required for interaction with the Ccr4-Not complex.

Previously, we TAP-purified individual Ccr4-Not subunits and identified associated proteins by mass spectrometry [TAP purification gels previously published in 41,49]. Among the proteins which co-purified with multiple Ccr4-Not subunits and which were not previously reported were the S-adenosylmethionine synthetases, Sam1 and Sam2, ( 1, Table S1, Figure S1), enzymes that regulate the cellular pool of S-adenosylmethionine (SAM) which is the universal methyl donor required for numerous biochemical reactions including Hmt1-dependent protein methylation [50]. This association between Ccr4-Not subunits and the Sam proteins is consistent with a role for Ccr4-Not in Hmt1-mediated protein methylation.

**Table 1:** Ccr4-Not TAP purifications identify Mlp1/2 and Sam1/2.

Ccr4-Not TAP Purification	Co-purified protein
*CAF40-TT*	Mlp1 (6)^a^, Mlp2 (14), Sam1 (27), Sam2 (27)
*CAF40-TT caf130Δ*	Mlp1 (27), Mlp2 (11), Nup60 (13), Mft1 (30)
*CAF40-TT not3Δ*	Mft1 (23), Sam1 (27)
*CAF130-TT*	Mlp1 (39)
*NOT1-TT caf40Δ*	Mlp1 (45)
*NOT2-TT*	Mlp1 (26), Mft1 (30) Sam1 (29)
*NOT2-TT caf40Δ*	Mft1 (42)
*NOT3-TT caf40Δ*	Mft1 (36)
*NOT4-TT*	Mlp2 (27), Sam1 (35), Sam2 (20)

a MASCOT scores are presented in parentheses next to each identified protein.

### Components of Ccr4-Not physically and functionally interact with the nuclear pore complex and mRNA processing and export factors

**Figure 3:**
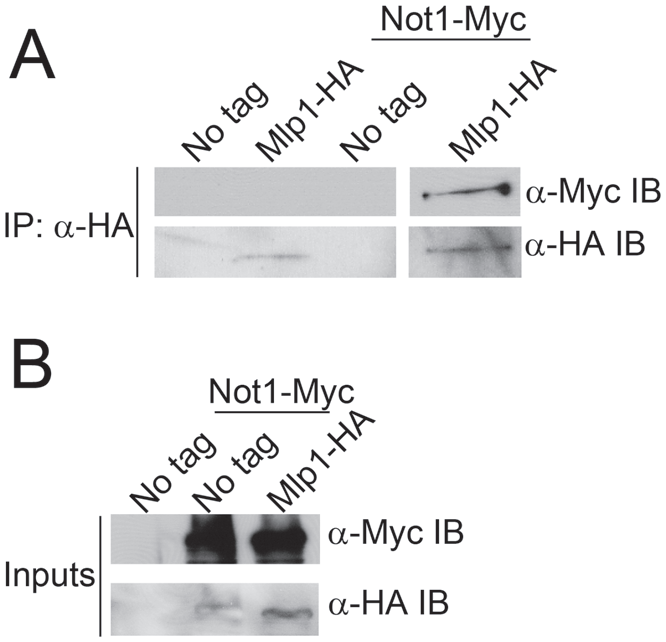
Not1 co-associates with the nuclear basket subunit Mlp1. (A) Whole-cell extracts from cells individually expressing *NOT1-Myc* or *MLP1-HA* or from cells expressing both tagged alleles were used in α-HA immunoprecipitation experiments to pull-down Mlp1. Co-associated Not1 was detected by α-Myc immunoblot which was subsequently stripped and reprobed with α-HA to detect immunoprecipitated Mlp1. (B) Input samples (30 µg) were initially probed with α-Myc to detect Not1 levels and then the membrane was stripped and reprobed with α-HA to detect Mlp1.

In multiple independent TAP purifications of Ccr4-Not subunits analyzed on SDS-PAGE or native gels followed by either one of two different mass spec analyses we identified the Mlp1, Mlp2, Nup2, and Nup60 components of the inner nuclear basket of the NPC [51], [52], [53]. We also identified the mRNA processing and export factors Dbp5, Mft1, and Yra1 [54], [55], [56], [57], [58]. Tables 1 and 2 present the list of proteins or peptides identified as co-purifying with Ccr4-Not subunits and their MASCOT scores. Identified peptides for each purification are presented in Tables 2 and S1, and representative gel images are presented in Figure S1. Though many of the MASCOT scores in the first analysis (Table 1) are relatively low, these scores are similar to the scores obtained for some of the Ccr4-Not subunits isolated in the purifications and thus most likely reflect the richness and complexity of the individual purifications. Furthermore, the analyses performed subsequently with a more powerful machine identified with 100% confidence the indicated co-purifying proteins (Table 2). To further validate the association of Ccr4-Not members specifically with the NPC, we performed co-immunoprecipitation experiments from cell extracts containing Not1-Myc and Mlp1-HA epitope tags. Immunoprecipitation of Mlp1 readily co-precipitated Not1 in these experiments, thus further confirming our mass spectrometry results and supporting the observation that Ccr4-Not associates with the NPC (Figure 3).

**Table 2:** Ccr4-Not TAP purifications identify Mlp2 and mRNA processing and export factors.

Ccr4-Not TAP Purification	Co-purified protein	Protein identification probability	Peptide sequence	Best peptide identification probability	Best MASCOT ion score	Best MASCOT identity
*NOT2-TT caf130Δ*	Yra1Nup2Dbp5	100%100%99.8%	AVERFNGSPIDGGREFFASQVGGVQRLNLIVDPNQRPVKSLDEIIGSNKAGSNRALNLQFKKTETNAKPFSFSSATSTTEQTKMAFKPFGSAKSDETKSKVLITTNVLARVLITTNVLAR	95%95%95%95%95%95%95%95%95%	41.660.34269.142.855.132.231.665	25.524.616.722.323.221.522.913.818.3
*NOT5-TT caf130Δ*	Mlp2	100%	DAIIELENINAKFLDQNSDASTLEPTLRKLLASTEENKANTNSVTSMEAAR	95%95%95%	37.933.663.2	22.923.621.1

As Mlp1, Mlp2, and Nup60 are all nuclear NPC components, their co-purification with Ccr4-Not subunits suggests that interactions between Ccr4-Not and the NPC may occur on the nuclear face of the NPC, a subnuclear domain that has critical roles in mRNA export [4], [5]. Moreover, the identification of the THO complex subunit, Mft1, as well as Yra1 and Dbp5, provide additional evidence for a link between Ccr4-Not and mRNA processing and export. Given the role of the Mlp proteins in mRNA export and quality control and their interactions with mRNA binding proteins [59], [60], [61], we tested whether interactions between the Mlp proteins and Ccr4-Not subunits may be RNA-dependent. However, RNase treatment did not decrease the interactions between Mlp1 and Caf1 (data not shown), indicating that these interactions are not mediated by RNA.

**Figure 4:**
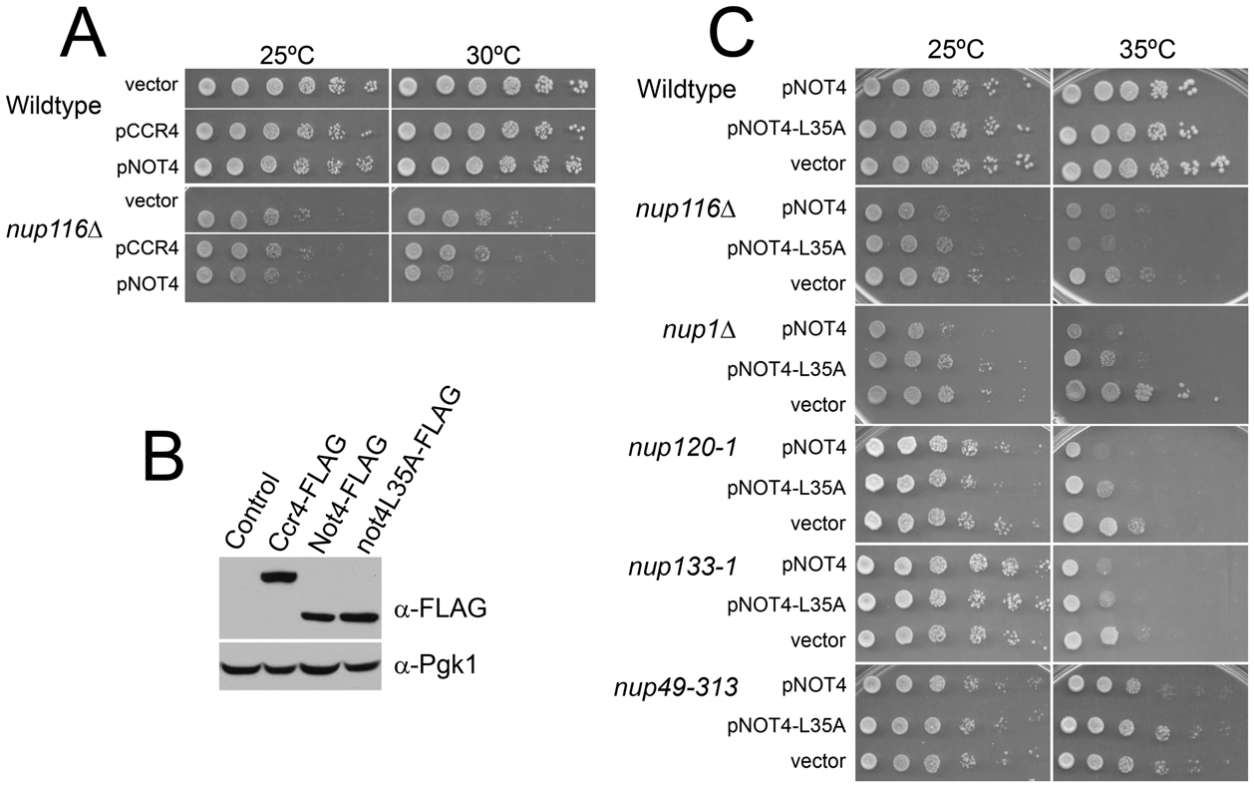
Not4 and not4L35A overexpression cause differential growth effects in NPC mutant cells. (A) Wild-type and *nup116Δ* cells were transformed with empty vector, *CCR4* or *NOT4* overexpression constructs. Cells were grown to saturation in SC-Ura media, ten-fold serially diluted, and spotted onto SC-Ura plates. Plates were incubated at 25°C or 30°C. (B) Whole cell extracts of cells transformed with empty vector, *CCR4*, *NOT4*, or *not4L35A* were analyzed by immunoblotting and probed with α-FLAG antibody. Blots were probed with α-PGK1 antibody as a loading control. (C) Wildtype, *nup1Δ*, *nup116Δ*, *nup120-1*, *nup133-1*, and *nup49-313* cells were transformed with empty vector, *NOT4*, or *not4L35A* overexpression constructs. Cells were grown to saturation in SC-Ura media, ten-fold serially diluted, and spotted onto SC-Ura plates. Plates were incubated 25°C or 35°C.

To determine the functional relevance of Ccr4-Not association with the NPC, we tested for genetic interactions between the catalytically active Ccr4-Not subunits: the major deadenylase, Ccr4, and the E3 ubiquitin ligase, Not4, and various NPC components. Gene deletions of several Ccr4-Not complex members result in profound growth defects [26], [27]; therefore, we assayed for genetic interactions by overexpressing these Ccr4-Not subunits in cells deleted for *NUP116*, a NPC component implicated in mRNA export [62]. Interestingly, these experiments revealed that overexpression of *NOT4*, but not *CCR4*, impairs growth of *nup116Δ* cells (Figure 4A). This differential effect is not due to differences in overexpression levels between Not4 and Ccr4, as immunoblotting confirms that both proteins are overexpressed to similar levels (Figure 4B). As a control, overexpression of Ccr4-Not subunits has no effect on the growth of wild type cells (Figure 4A).

Not4 encodes a ubiquitin E3 ligase whose *in vivo* substrates remain largely unknown [31], [32], [33], [63]. To determine if the Not4 ligase function is important for the overexpression phenotype in *nup116Δ* cells and to extend our functional analysis to other NPC subunits, we overexpressed *NOT4* and the *not4L35A* mutant in *nup116Δ* cells and a variety of other temperature sensitive NPC gene deletions or mutants. The *not4L35A* mutation disrupts interactions between Not4 and its two known E2 ubiquitin-conjugating enzymes, Ubc4 and Ubc5, thus compromising its ubiquitin ligase function [63]. *NOT4* overexpression causes significant growth defects in cells individually mutated or deleted for several NPC components, including *nup1Δ*, *nup116Δ*, *nup120-1*, and *nup133-1* mutant cells (Figure 4C). In contrast, *NOT4* overexpression only modestly affected *nup49-313* mutant cells and showed no effect on wild-type cells. Interestingly, overexpression of *not4L35A* mostly mirrored the effect of *NOT4* overexpression except that it had a slightly less negative effect on growth in *nup1Δ* and *nup120-1* cells and no detectable effect in *nup49-313* cells (Figure 4C). These effects are not due to differences in the level of overexpression between Not4 and not4L35A, as immunoblotting confirms that both proteins are overexpressed to similar levels (Figure 4B). The differences in the effects of *NOT4* and *not4L35A* overexpression in the *nup1Δ*, *nup120-1*, and *nup49-313* cells are not completely surprising as *not4Δ* and *not4L35A* mutants have both overlapping and distinct phenotypic effects [63]. Taken together, these results suggest that altered stoichiometry of the Not4 ligase is detrimental for cells with compromised NPCs, and that these negative growth effects are only partially dependent on Not4 interactions with Ubc4 and/or Ubc5.

### Not4 functionally interacts with hnRNPs

**Figure 5:**
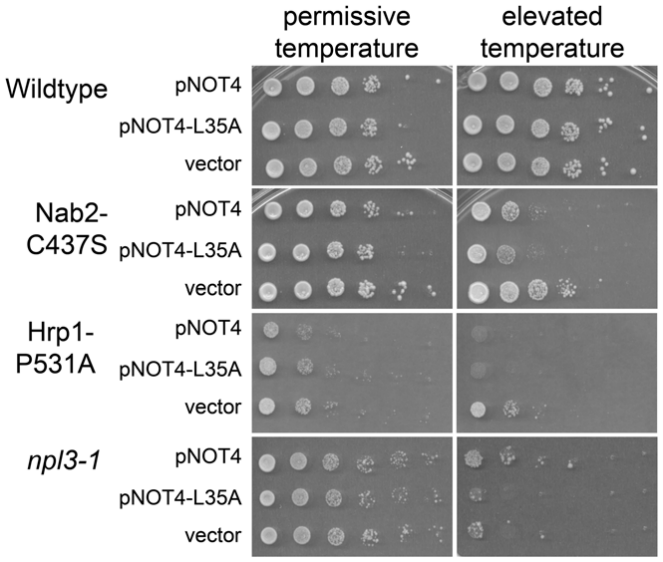
Not4 and not4L35A overexpression cause differential growth effects in hnRNP mutant cells. Wildtype, Nab2-C437S, Hrp1-P531A and *npl3-1* cells were transformed with empty vector, *NOT4* or *not4L35A* overexpression constructs. Cells were grown to saturation in SC-Ura media, ten-fold serially diluted, and spotted onto SC-Ura plates. Plates were incubated at 25°C for the permissive temperature or 39°C (for Nab2-C437S) 37°C (for Hrp1-P531A), or 30°C (for *npl3-1*) for the elevated temperature.

Given the importance of Hmt1-mediated methylation of hnRNPs for efficient hnRNP nuclear export [44], [64], and the critical role of the NPC in mRNA export, we next examined whether Ccr4-Not associations with hnRNPs and NPC components might have functional implications for mRNA nuclear export. To investigate this question, we tested for genetic interactions between Not4 and various hnRNPs by overexpressing *NOT4* and *not4L35A* in a variety of hnRNP mutants, some of which have mRNA export defects [19], [20], [21], [22]. Intriguingly, this analysis revealed that overexpression of *NOT4* is deleterious to cells expressing mutant versions of Nab2 and Hrp1 but not to wild-type cells (Figure 5). In sharp contrast, overexpression of *NOT4* weakly suppresses the severe temperature-sensitive growth phenotype of the *npl3-1* mutant (Figure 5). Similar to results in NPC mutants, overexpression of *not4L35A* had different effects on growth in different hnRNP mutants. Overexpression of *not4L35A* had deleterious effects on Nab2 and Hrp1 mutants similar to effects of wildtype *NOT4* overexpression, whereas *not4L35A* overexpression did not suppress the temperature sensitivity of the Npl3 mutant (Figure 5). These results suggest that the Not4 ubiquitin ligase has both ligase-dependent and independent interactions with hnRNPs and NPC mutants essential for mRNA export.

### NOT4 overexpression exacerbates the poly(A) export defect in a nuclear pore mutant

**Figure 6:**
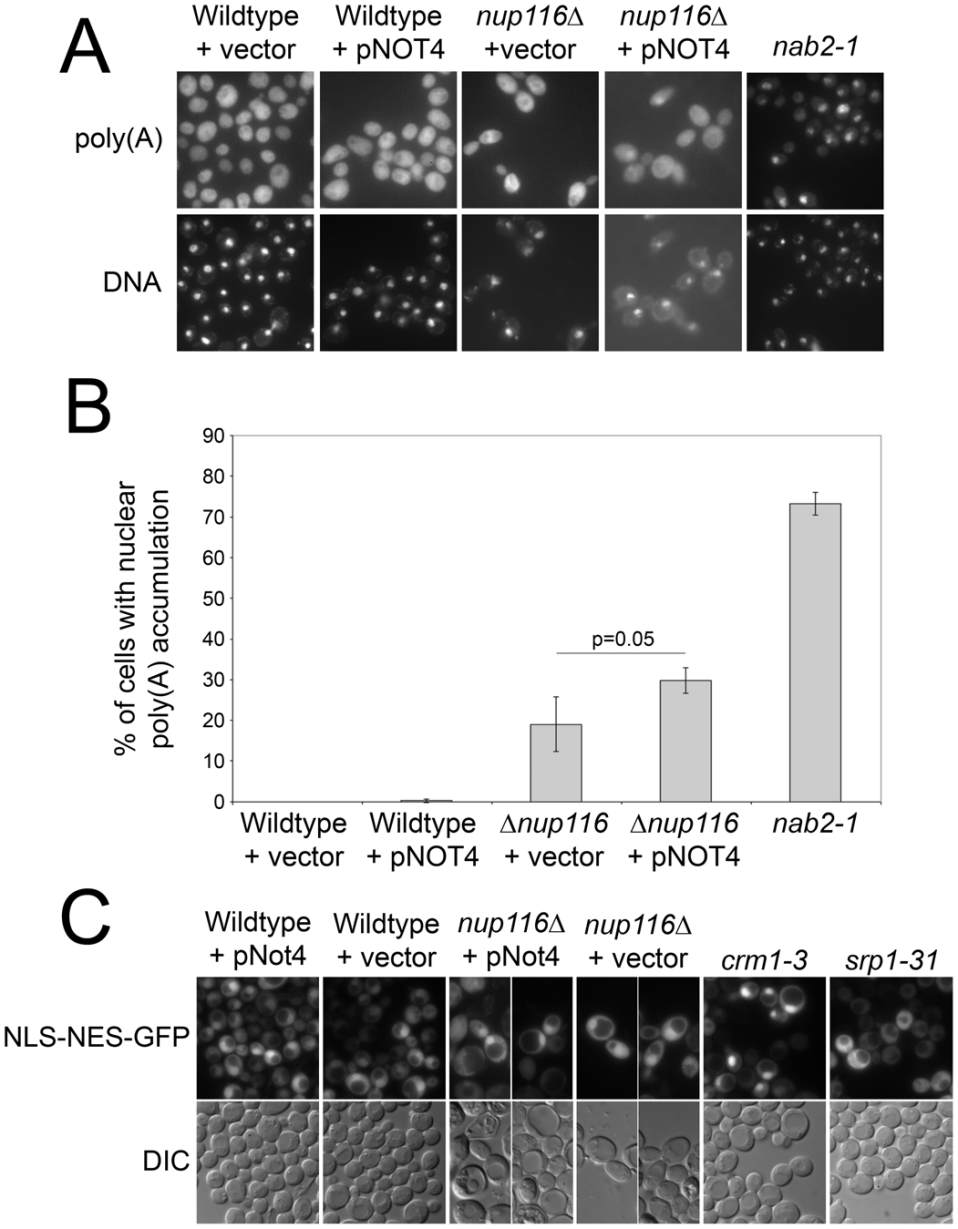
Not4 overexpression exacerbates the poly(A) RNA export defect in *nup116Δ* cells. Wildtype and *nup116Δ* cells were transformed with empty vector or a *NOT4* overexpression construct. Cells were grown to log phase at 30°C and subjected to FISH. (A) FISH was performed on cells as described in Materials and Methods. Panels are shown for poly(A) RNA and DAPI to visualize chromatin. (B) Quantification of cells showing nuclear accumulation of poly(A) RNA. Images were analyzed blind, and a minimum of 50 cells were analyzed in triplicate for each condition. Student's *t*-test was used to determine statistical significance. (C) Wildtype and *nup116Δ* cells were transformed with empty vector or a *NOT4* overexpression construct and an NLS-NES-GFP construct. Cells were grown to log phase and analyzed by live cell microscopy for GFP localization.

The results presented above suggest that Ccr4-Not may have an unrealized role in the nuclear mRNA processing and export pathway. This hypothesis is supported through the physical associations between Ccr4-Not members and both hnRNPs and the NPC and also by the functional interactions between Not4 and many of the temperature sensitive NPC mutants (see Figure 4C) that have poly(A) RNA export defects at the nonpermissive temperature [19], [20], [21], [22]. We initially assayed for defects in global mRNA export in wild-type and various Ccr4-Not deletion mutants by fluorescence *in situ* hybridization (FISH), but did not detect significant mRNA nuclear accumulation in any of these mutants (data not shown). Because the mRNA export pathway is highly robust and redundant, we speculated that inhibition of Ccr4-Not alone may not result in a detectable defect in global mRNA export. Deletion of *NUP116* results in nuclear accumulation of bulk poly(A) RNA at 37°C [22]. The growth defects we observed in *nup116Δ* cells overexpressing *NOT4* (see Figure 4A) suggested the possibility that NOT4 overexpression might exacerbate the mRNA export defect in these cells. To investigate this possibility, we conducted FISH analysis to detect bulk poly(A) mRNA localization in cells deleted for *NUP116* and overexpressing the *NOT4* gene. This analysis revealed a modest but statistically significant increase (p = 0.05) in nuclear poly(A) RNA accumulation in *nup116Δ* cells overexpressing *NOT4* (29.84±2.70%) compared to *nup116Δ* carrying control plasmid (19.00±6.16%) (Figure 6A, 6B). As controls, wild-type cells overexpressing *NOT4* showed no increase in nuclear poly(A) RNA signal whereas the *nab2-1* mutant results in significant accumulation of poly(A) RNA (73.17±2.79%), consistent with previous reports [45].

In order to determine whether this nuclear RNA accumulation is due to a general defect in nucleocytoplasmic transport, we expressed a GFP reporter fused to a nuclear localization signal (NLS) and a nuclear export signal (NES) (NLS-NES-GFP) [65] in *nup116Δ* overexpressing *NOT4*. This analysis revealed no apparent difference in the localization of the NLS-NES-GFP construct in *nup116Δ* cells overexpressing *NOT4* compared to control cells containing empty vector (Figure 6C). Wildtype cells overexpressing *NOT4* also show no apparent difference in NLS-NES-GFP localization compared to cells containing empty vector. As controls, cells mutant for the export factor, Crm1 (*crm1-3*) [66], display nuclear accumulation of NLS-NES-GFP, whereas cells mutant for the import factor, Srp1 (*srp1-*31) [67], [68], show a cytoplasmic distribution of NLS-NES-GFP compared to wildtype cells. These results indicate that the nuclear RNA accumulation observed in *nup116Δ* cells overexpressing *NOT4* is not due to a general disruption in nucleocytoplasmic trafficking. Taken together, these results suggest a functional relationship between Ccr4-Not, components of the mRNA export pathway, and the NPC. Moreover, these results demonstrate that steady-state mRNA export is susceptible to alterations in Ccr4-Not when combined with NPC mutants.

## Discussion

Our results identify new connections between the Ccr4-Not complex and the mRNA processing and export machinery through physical and functional interactions both with hnRNPs and the NPC. The identification of Sam1 and Sam2 in our Ccr4-Not TAP-purifications further supports the hypothesis that Ccr4-Not likely plays an important role in protein methylation. This effect most likely does not involve global regulation of Hmt1-dependent methylation, however, since no effect on Npl3 methylation was detected in Ccr4-Not mutant cells. We also present the novel finding that altered stoichiometry of the Ccr4-Not complex, specifically by increased expression of the Not4 ubiquitin ligase, causes significant growth defects in cells mutant for Nab2, Hrp1, and NPC subunits. In stark contrast, we find that Not4 overexpression rescues growth of the *npl3-1* mutant at the non-permissive temperature. Furthermore, we demonstrate that increased Not4 expression modestly exacerbates the mRNA export defect seen in *nup116Δ* cells, further suggesting that the Ccr4-Not complex plays a functional role in mRNA processing and export. This point is further supported by the co-purification of Mlp1 and Mlp2 proteins with Ccr4-Not members, including Not4. These results significantly extend previous findings from high throughput screens which identified a putative physical interaction between the Ccr4-Not complex and the mRNA export factor Yra1 and a putative genetic interaction between the Ccr4-Not complex and Nup116 [69], [70]. Recently, the human Ccr4-Not complex was purified and a number of mRNA export and nuclear pore proteins were found to co-purify with the complex [71]. Our results, in combination with the analysis of human Ccr4-Not strongly suggest that associations between Ccr4-Not and components of the mRNA export pathway and nuclear pore are evolutionarily conserved and functionally relevant. Taken together, these results expand the functions of the Ccr4-Not complex in the lifecycle of an mRNA from its known roles in transcriptional regulation and mRNA degradation to newly identified connections to mRNA export.

The initial report demonstrating that hCaf1 interacts with PRMT1 and regulates its methyltransferase activity suggested that Ccr4-Not plays a role in PRMT1-dependent processes [42]. However, the *in vivo* relevance of these interactions was not explored in detail. We augment these preliminary findings by demonstrating that multiple components of the budding yeast Ccr4-Not complex associate with the PRMT1 homolog, Hmt1, and that Ccr4-Not also associates with the Hmt1 substrates, Hrp1 and Nab2 in an Hmt1 methyltransferase-dependent fashion [44], [45]. The finding that interactions between Ccr4-Not components and these hnRNPs depend on Hmt1 methyltransferase activity strongly suggests that Ccr4-Not predominantly interacts with methylated, export-competent hnRNPs in an RNA-independent manner.

The Ccr4-Not complex has recently been implicated in nuclear RNA quality control through interactions with the TRAMP complex and nuclear exosome [41]. In addition, we demonstrate that Ccr4-Not co-purifies with components of the NPC nuclear basket including Mlp1 and Mlp2, which have a well established role in mRNA export quality control [59], [60] as well as Mft1, Yra1, and Dbp5 which play different roles in mRNA processing and export. These results extend the previous findings of physical and functional interactions between the Ccr4 subunit of Ccr4-Not and the Hpr1 subunit of the THO complex [38]. The observation that Ccr4-Not interacts both with methylated, export-competent hnRNPs and NPC nuclear basket components is consistent with a model in which Ccr4-Not selectively interacts with methylated hnRNPs as they chaperone their mRNA cargoes through the NPC. The demonstration that Hmt1 methyltransferase activity is required for Ccr4-Not to associate with hnRNPs, coupled with the identification of Mft1, Yra1, Sam1, and Sam2 as Ccr4-Not co-purifying factors, suggests that Ccr4-Not may act to physically position these factors at the NPC to facilitate methylation and subsequent nuclear mRNA export. Our genetic analysis demonstrating that increased expression of the Not4 ubiquitin ligase results in synthetic growth defects in cells mutant for Nab2, Hrp1, and NPC subunits suggests that Ccr4-Not also may have other, as yet undefined roles in the mRNA export pathway that become dysregulated when Not4 exists in excess. This hypothesis is supported by our results demonstrating that Not4 overexpression exacerbates the mRNA export defect in *nup116Δ* cells. Interestingly, Not4 overexpression is not universally detrimental to hnRNP mutants, as it rescues the extreme temperature sensitivity of *npl3-1* cells which also display mRNA processing and export phenotypes [14], [72], [73]. These differential effects of Not4 overexpression suggest a complex and nuanced interaction between different components of the mRNA export pathway. As ubiquitination has been implicated in control of mRNA processing and export [74], [75], [76], our experiments raise the possibility that some of these factors may be targets of the Not4 ubiquitin ligase. This potential activity of Not4 toward mRNA processing and export factors may regulate modification of their function through mono-ubiquitination or target them for degradation through poly-ubiquitination.

We demonstrate that the Not4L35A mutant, which blocks interaction with the ubiquitin conjugating enzymes Ubc4 and Ubc5 (Ubc4/5) [63], has differential effects with different hnRNP and NPC mutants relative to wild-type Not4. These effects suggest the possibility that Ccr4-Not may be part of a complex regulatory cascade that partially depends on interactions between Not4 and Ubc4/5. Interestingly, Ubc4/5 also interact with the Tom1 ubiquitin ligase, a HECT-domain ligase [77] whose E3 ligase function is implicated in mRNA export [74]. One possible mechanism by which Ccr4-Not might regulate mRNA export is through interactions with Ubc4/5, which might reduce or prevent Ubc4/5 interactions with Tom1 and thus prevent Tom1-mediated ubiquitination of downstream targets. In addition, it is possible that as yet unidentified targets of Not4 may play a role in mRNA export control. This possibility is consistent with the fact that deletion of *NOT4* results in significant growth defects that are not phenocopied by deletion of known substrates [32], [33], suggesting that a number of important Not4 targets remain to be identified. In addition to its activity as a ubiquitin ligase, Not4 also contains a putative RNA recognition motif (RRM), that has significant sequence similarity to characterized RNA binding domains [26], [78]. Although the *in vivo* significance of this domain is unknown, it is possible that Ccr4-Not may bind RNA *via* the Not4 subunit as part of its role in mRNA export. One speculative possibility is that the Not4 overexpression phenotype in cells mutant for hnRNPs and NPC subunits may result from dysregulated interactions of Not4 with specific mRNA classes. This possibility could explain the modest mRNA export phenotype seen in *nup116Δ* cells since only a sub-population of mRNAs may be preferentially retained in the nucleus under these conditions. However, whether Not4 binds RNA *in vivo*, and the detailed mechanism by which it affects the mRNA processing and export machinery to impact mRNA export, remain to be addressed in future studies. As the Ccr4-Not complex and the nuclear pore are both large complexes with many subunits, it will take significantly greater understanding of the architecture of each complex in order to analyze the molecular details underlying their physical and functional interactions.

## Materials and Methods

### Strains, Plasmids, and Chemicals

All DNA manipulations were performed according to standard methods [79] and all media were prepared by standard procedures [80]. All *S. cerevisiae* strains and plasmids used are described in Table 3. Plasmid pAC2668 was generated by using the QuikChange Site-Directed Mutagenesis (Stratagene, La Jolla, CA) approach and plasmid pAC2492 (*NOT4*) as template. The resulting mutation was confirmed by sequence analysis. All chemicals were obtained from Ambion (Austin, TX), Sigma Chemical Co. (St. Louis, MO), US Biological (Swampscott, MA) or Fisher Scientific (Pittsburgh, PA) unless otherwise noted.

**Table 3:** Strains and plasmids used in this study.

Strain	Description	Reference/Source
FY23 (ACY192)	*MATa ura3 leu2 trp1*	[84]
BY4741 (ACY402)	*MAT a his3 leu2 met15 ura3*	Open Biosystems
LDY561 (ACY786)	*MAT α nup1Δ::LEU2 ura3 leu2 trp1 his3 ade3*	[85]
SWY27 (ACY542)	*MAT α nup116Δ::HIS3 ura3 leu2 trp1 his3 ade2 can1*	[22]
Dat4-2 (ACY1136)	*MAT a nup120-1 ura3 leu2 trp1*	[86]
Dat3-2 (ACY1135)	*MAT a nup133-1 ura3 leu2 trp1*	[86]
ACY1903	*nup49Δ::KANMX4 ura3 his3 leu2* (pUN90-LEU2-nup49-313)	This study
ACY427	*MAT a nab2Δ::HIS3 ura3 leu2 his3* (pAC636)	[87]
SVL182/PSY1224 (ACY1571)	*hrp1Δ HIS3 ura3 his3* [*HRP1 CEN URA3*]	S.R. Valentini
ACY71	*MATα npl3-1 trp1 ura3 leu2*	M. Henry
H3247	*Mat a his3 leu2 met15 ura3 CAF1-MYC13::HIS3 MX6*	[88]
H3239	*Mat a his3 leu2 met15 ura3 CCR4-MYC13::HIS3 MX6*	[88]
H2341	*Mat a his3 leu2 met15 ura3 NOT2-MYC13::HIS3 MX6*	[88]
H3245	*Mat a his3 leu2 met15 ura3 NOT5-MYC13::HIS3 MX6*	[88]
YNL052	*Mat a his3 leu2 met15 ura3 NOT1-MYC13::HIS3 MX6*	This study
YNL068	*Mat a his3 leu2 met15 ura3 HMT1-6XHA::KANMX4*	This study
YNL069	*Mat a his3 leu2 met15 ura3 CCR4-MYC13::HIS MX6 HMT1-6XHA::KANMX4*	This study
YNL070	*Mat a his3 leu2 met15 ura3 NOT2-MYC13::HIS MX6 HMT1-6XHA::KANMX4*	This study
YNL071	*Mat a his3 leu2 met15 ura3 NOT5-MYC13::HIS MX6 HMT1-6XHA::KANMX4*	This study
YNL081	*Mat a his3 leu2 met15 ura3 NOT1-9XMYC::hphNT1*	This study
YNL138	*Mat a his3 leu2 met15 ura3 MLP1-6XHA::KANMX4*	This study
YNL139	*Mat a his3 leu2 met15 ura3 MLP1-6XHA::KANMX4 NOT1-9XMYC::HPHNT1*	This study
YNL179	*Mat a his3 leu2 met15 ura3 CAF1-MYC13::HIS3 MX6 hmt1Δ::KANMX4*	This study
PSY1221 (ACY339)	*Mat a ade2 ade3 his3 trp1 ura3 leu2 crm1-3*	[66]
ACY1561	*Mat a ura3 leu2 trip1 his3 lys2 spr1-31*	[68]
MY4858	*MAT α leu2 ura3 met15 his3 caf40::CAF40-TAP-URA3*	[49]
MY4985	*MAT a leu2 ura3 met15 his3 caf40::CAF40-TAP-URA3 caf130Δ::HIS3*	[35]
MY4980	*MAT α leu2 ura3 met15 his3 caf40::CAF40-TAP-URA3 not3Δ::HIS3*	[35]
MY4857	*MAT α leu2 ura3 met15 his3 not4::NOT4-TAP-URA3*	[41]
MY5218	*MAT α leu2 ura3 met15 his3 caf130::CAF130-TAP-KANMX4*	This study
MY5026	*MAT a leu2 ura3 met15 his3 not2::NOT2-TAP-KANMX4*	[41]
MY5711	*MAT a leu2 ura3 met15 his3 not2::NOT2-TAP-KANMX4 caf40Δ::HIS3*	[41]
MY5273	*MAT α leu2 ura3 met15 his3 not2::NOT2-TAP-KANMX4 caf130Δ::HIS3*	This study
MY7079	*MAT a leu2 ura3 met15 his3 not1::NOT1-TAP-URA3 caf40Δ::HIS3*	This study
MY6013	*MAT a leu2 ura3 met15 his3 not3::NOT3-TAP-KANMX4 caf40Δ::HIS3*	This study
MY5892	*MAT a leu2 ura3 met15 his3 not5::NOT5-TAP-KANMX4 caf130Δ:: KANMX4*	This study

Plasmids	Description	Reference/Source
pN827	*pADH1 CEN URA3*	[89]
pAC2492	*pADH1-NOT4-FLAG CEN URA3*	[37]
pAC2494	*pADH1-CCR4-FLAG CEN URA3*	This Study
pAC2668	*pADH1-not4-L35A-FLAG CEN URA*	This Study
pUN90-LEU2-nup49-313	*nup49-313 CEN LEU2*	[19]
pAC636	*NAB2 CEN URA3*	[45]
pSW3298 (pAC2307)	*nab2-C437S CEN LEU2*	[90]
pAC2539	*hrp1-P531A CEN LEU2*	[87]
pPS1750 (pAC2813)	*hmt1-G68R CEN URA3*	[46]
pPS1307 (pAC2811)	*HMT1 CEN URA3*	[43]
pRS306 (pAC4)	*CEN URA3*	[91]
pAC212	*pADH1-NLS-NES-GFP 2 µm TRP1*	[65]

### Tandem-affinity purification (TAP)

TAP-purifications listed in Tables 1 and 2, Figure S1 and Table S1 were performed and analyzed as previously described [41], [49]. Briefly, yeast strains expressing TAP-tagged subunits were grown to log phase and whole cell extracts were prepared. Purified proteins were resolved on 4-12% SDS-PAGE or 3–12% native (Invitrogen) gradient gels and stained with Coomassie. Detectable bands were excised from the gel, and MALDI-TOF mass spectrometry analysis of the excised bands was performed for the initial SDS-PAGE gels, or NanoLC-ESI-MS/MS for the native gel and subsequent SDS-PAGE bands, at the Proteomics Core Facility of the Faculty of Medicine, University of Geneva. For the MALDI-TOF analysis, the spectra obtained were analyzed using the DATA EXPLORER program and proteins identified using the MASCOT SEARCH website. Table S1 lists the different Ccr4-Not subunits TAP-purified, the co-purified NPC factors, and both the peptide amino acid sequences and their respective positions in the identified proteins. For the NanoLC-ESI-MS/MS, the analysis was done with the Mascot program (Matrix Science, London, UK; version Mascot). Mascot was set up to search the uniprot_sptr_15.10-03-Nov-2009 database (selected for *Saccharomyces cerevisiae*, 34911 entries) assuming the digestion enzyme trypsin. Mascot was searched with a fragment ion mass tolerance of 0.60 Da and a parent ion tolerance of 10.0 ppm. Iodoacetamide derivative of cysteine and oxidation of methionine were specified as fixed and variable modifications, respectively. Scaffold program (version Scaffold 03, Proteome Software, Inc., Portland, OR) was used to validate the MS/MS based peptide and protein identifications. Peptide identifications were accepted if they could be established at greater than 95.0% probability as specified by the Peptide Prophet algorithm [81]. Protein identifications were accepted if they could be established at greater than 95.0% probability and contained at least 2 identified peptides. Protein probabilities were assigned by the Protein Prophet algorithm [82]. Proteins that contained similar peptides and could not be differentiated based on MS/MS analysis alone were grouped to satisfy the principles of parsimony.

### Co-immunoprecipitation experiments

Asynchronous cell cultures were grown to log phase before pelleting and lysing cells in immunoprecipitation buffer (10 mM Tris, pH 8.0, 150 mM NaCl, 0.1% Nonidet P-40, 10% glycerol containing protease and phosphatase inhibitors, 1 mM DTT) using bead beating as previously described [37]. Immunoprecipitations were performed using 1 mg of whole-cell extract (for experiments detecting Hmt1 or NPC associations) or 500 µg (for Hrp1 and Nab2 associations) and 2–3 µL of α-Myc or α-HA antibody (Santa Cruz Biotechnology). Immunoprecipitations were rotated 2 hours to overnight at 4°C before immune complexes were captured using Protein A-conjugated agarose (Santa Cruz Biotechnology). Samples were washed 3 times with 0.5–1 mL immunoprecipitation buffer and then resolved by SDS-PAGE and detected by immunoblot analysis. Nab2 and Hrp1 were detected by α-Nab2 and α-Hrp1 specific antibodies [[45] and gift from M. Swanson]. Bands were quantitated using reverse image scanning densitometry (Photoshop CS2, Adobe) by normalizing the band intensity of the co-immunoprecipitated protein to the band intensity of the immunoprecipitated protein. For RNase experiments, cell extracts from no tag control or cells expressing Caf1-Myc were prepared as described above. Extracts were either mock treated or treated with RNase A (0.105 U/µL) in 400 mL IP buffer for 10 min at 37°C before antibody addition. IP samples were rotated overnight at 4°C and the IP performed as described above.

### Fitness analysis

For serial dilution spotting assays, single colonies of wildtype or mutant cells expressing plasmid-borne *CCR4*, *NOT4*, *not4L35A*, or empty vector were grown to saturation in selective liquid culture lacking uracil (ura^-^), normalized to equal starting concentrations as assayed by optical density or cell counting by hemocytometer, and then serially diluted (1:10) in dH_2_O and spotted onto selective ura^-^ plates. Plates were incubated at 25, 30, 33, 35 or 37°C for 2–4 days as indicated.

### Microscopy

All microscopy was carried out using filters from Chroma Technology and an Olympus BX60 epifluorescence microscopy equipped with a Photometrics Quantix digital camera. For live cell microscopy, cells expressing NLS-NES-GFP [65] were grown to early log phase and GFP signal was detected through a GFP-optimized filter. Images were captured using IP Lab Spectrum software.

### Fluorescence *in situ* hybridization (FISH)

The intracellular localization of poly(A) RNA was assayed essentially as described [45], [83]. Briefly, cells were grown to saturation overnight at 25°C and subsequently diluted and incubated for 2 h to allow cells to re-enter growth phase. Cells were then shifted to 30°C for 2–4 h. Cells were fixed with 4.2% formaldehyde. The cell wall was digested with 0.5 mg/mL zymolase, and cells were applied to multi-well slides (Thermo Electron Corporation) pre-treated with 0.1% polylysine. Cells were then permeabilized with 0.5% NP-40, equilibrated with 0.1 M triethanolamine, pH 8.0, and incubated with 0.25% acetic anhydride to block polar groups. Cells were then incubated in prehybridization buffer (50% deionized formamide, 10% dextran sulfate, 4X Sodium Chloride-Sodium Citrate buffer (SSC), 1X Denhardt's solution, 125 µg/mL tRNA) and hybridized overnight with digoxigenin-labeled 50-mer oligo(dT) probe (IDT DNA). Wells were washed several times and blocked in 0.1 M Tris pH 9.0, 0.15 M NaCl, 5% heat-inactivated fetal calf serum, and 0.3% Triton X-100. Cells were incubated 2 h with fluorescein isothiocyanate (FITC)-conjugated α-digoxigenin antibody (1:200, Roche). Wells were then washed several times and stained with 1 µg/µL 4′,6-diamidino-2-phenylindole-dihydrochlorine (DAPI) to detect chromatin. Cells were mounted in antifade medium (0.1% *p*-phenylenediamine, 90% glycerol in phosphate-buffered saline). Slides were stored at −20°C until visualization by microscopy. For quantification of results, blinded images were analyzed for poly(A) nuclear signal using the ImageJ Cell Counter plugin. A minimum of 50 cells were analyzed in triplicate for each condition. Unpaired Student's *t*-test assuming unequal variance was used to determine statistical significance.

## Supporting Information

Figure S1 **NPC components and mRNA processing and export factors co-purify with Ccr4-Not subunits.** (A) Total protein extract from wildtype or *caf130Δ* cells expressing Tap-tagged Caf40, Not4, Caf130, or Not2 were subjected to tandem affinity purification. After separation by SDS-PAGE followed by Coomassie staining, the purified proteins were identified by mass spectrometry analysis as described in Materials and Methods (see Table S1). Identified co-purifying proteins are indicated to the right of the gel lanes. Molecular weight markers (MW) are indicated to the left of the gels in kDa. MASCOT scores for identified co-precipitating proteins are identified in the table below the gel images. (B) Total protein extract from *caf130Δ* cells expressing Tap-tagged Not2, or Not5 were subjected to tandem affinity purification. After separation by SDS-PAGE, excised protein bands were analyzed at the Proteomics Core Facility of the Faculty of Medicine, University of Geneva as described in Materials and Methods (See Table 2 and Table S1). Identified co-purifying proteins are indicated to the right of the gel lanes. Protein identification probability scores for identified co-precipitating proteins are indicated in the table below the gel images.
(TIF)

Table S1 **Peptide sequences of Ccr4-Not co-purified factors.**
(DOCX)
