# Supplementary figures and images for: The Ccr4-Not Complex Interacts with the mRNA Export Machinery
Source: PLoS One. 2011 Mar 28;6(3):e18302. doi: 10.1371/journal.pone.0018302 (PMC3065485; doi:10.1371/journal.pone.0018302)

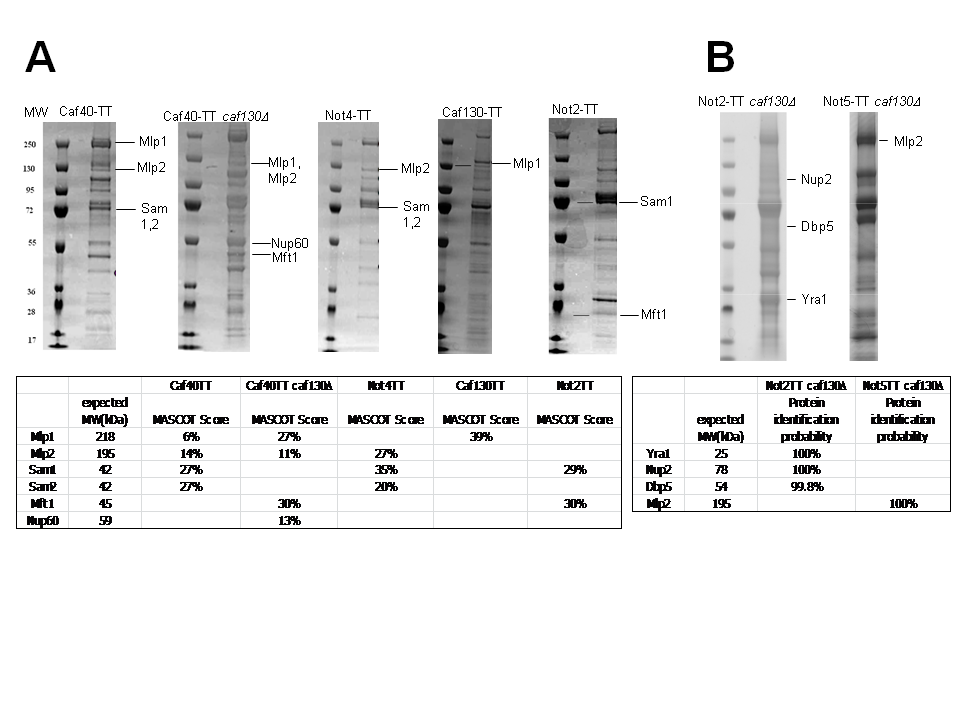

Supplement: Figure S1 — NPC components and mRNA processing and export factors co-purify with Ccr4-Not subunits. (A) Total protein extract from wildtype or caf130Δ cells expressing Tap-tagged Caf40, Not4, Caf130, or Not2 were subjected to tandem affinity purification. After separation by SDS-PAGE followed by Coomassie staining, the purified proteins were identified by mass spectrometry analysis as described in Materials and Methods (see Table S1). Identified co-purifying proteins are indicated to the right of the gel lanes. Molecular weight markers (MW) are indicated to the left of the gels in kDa. MASCOT scores for identified co-precipitating proteins are identified in the table below the gel images. (B) Total protein extract from caf130Δ cells expressing Tap-tagged Not2, or Not5 were subjected to tandem affinity purification. After separation by SDS-PAGE, excised protein bands were analyzed at the Proteomics Core Facility of the Faculty of Medicine, University of Geneva as described in Materials and Methods (See Table 2 and Table S1). Identified co-purifying proteins are indicated to the right of the gel lanes. Protein identification probability scores for identified co-precipitating proteins are indicated in the table below the gel images. (TIF) [file pone.0018302.s001.tif]
