# Supplementary material for: The Ccr4-Not Complex Interacts with the mRNA Export Machinery
Source: PLoS One. 2011 Mar 28;6(3):e18302. doi: 10.1371/journal.pone.0018302 (PMC3065485; doi:10.1371/journal.pone.0018302)
Supplement: Table S1 — Peptide sequences of Ccr4-Not co-purified factors. (DOCX) [file pone.0018302.s002.docx]

Table S1. **Peptide sequences of Ccr4-Not co-purified factors.**

| **Ccr4-Not TAP-tag (TT)** | **Co-purified Factor** | **Co-purified Factor Amino Acid Residues** | **Peptide Sequence** |
| --- | --- | --- | --- |
| *CAF40-TT* | Mlp1 | 50-57 | LLQFNELK |
|  |  | 126-134 | KLDDLTEEK |
|  |  | 127-135 | LDDLTEEKK |
|  |  | 768-777 | QELNKLSPEK |
|  |  | 782-792 | IMVTQLQTLQK |
|  |  | 1605-1617 | DVPHSSHISDDER |
|  |  | 83-94 | TEMENVIRENDK |
|  |  | 778-792 | DSLRIMVTQLQTLQK |
|  |  | 1599-1617 | LQEELKDVPHSSHISDDER |
|  |  | 1375-1394 | IEELQNAKVAQGNNQLEAIR |
|  |  | 302-321 | LVDLLESQLNAVKEELNSIR |
|  |  | 969-987 | ISLLKEQMFNLNNELDLQK |
|  |  |  |  |
|  | Mlp2 | 1399-1405 | IIDERTK |
|  |  | 278-284 | NMNDLLR |
|  |  | 285-292 | SQLTSLEK |
|  |  | 982-988 | MIEKIEK |
|  |  | 592-600 | LLASTEENK |
|  |  | 828-836 | SLLTELSNK |
|  |  | 974-981 | NEVERIQK |
|  |  | 1448-1456 | IKEAEENLK |
|  |  | 619-628 | ELEAELSSTK |
|  |  | 1292-1301 | QAHEKLDASK |
|  |  | 843-852 | LSSEIENLDK |
|  |  | 880-889 | ELEQIQVQLK |
|  |  | 1272-1285 | LSSAENANADLENK |
|  |  | 670-681 | ERMLEEAIDHLK |
|  |  | 1516-1531 | GNSERPSAVAGFINQK |
|  |  | 1356-1369 | DTYRTLMEEIESLK |
|  |  | 672-686 | MLEEAIDHLKAELEK |
|  |  | 174-188 | YDTSVQEKELMLQSK |
|  |  | 837-852 | ETTIEKLSSEIENLDK |
|  |  | 863-879 | FLDQNSDASTLEPTLTK |
|  |  | 809-825 | DSQLKWAQNTIDDTEMK |
|  |  | 220-242 | LYQMQSNYESVFTYNKFLLNQNK |
|  |  |  |  |
|  | Sam1 | 41 - 61 | VACETAAKTGMIMVFGEITTK |
|  |  | 49 - 61 | TGMIMVFGEITTK |
|  |  | 49 - 61 | TGMIMVFGEITTK |
|  |  | 77 - 89 | IGYDDSAKGFDYK |
|  |  | 229 - 236 | YFIQPSGR |
|  |  | 237 - 252 | .FVIGGPQGDAGLTGRK |
|  |  | 301 - 327 | VQVQFSYAIGIAEPLSLHVDTYGTATK |
|  |  |  |  |
|  | Sam2 | 43 - 63 | VACETAAKTGMIMVFGEITTK |
|  |  | 51 - 63 | TGMIMVFGEITTK |
|  |  | 51 - 63 | .TGMIMVFGEITTK.A |
|  |  | 79 - 91 | IGYDDSAKGFDYK |
|  |  | 231 - 238 | YFIQPSGR |
|  |  | 239 - 254 | FVIGGPQGDAGLTGRK |
|  |  | 303 - 329 | VQVQFSYAIGIAEPLSLHVDTYGTATK |
|  |  | 43 - 63 | VACETAAKTGMIMVFGEITTK |
|  |  |  |  |
| *CAF40-TT caf130Δ* | Mlp1 | 1062-1068 | EQLHTYK |
|  |  | 1110-1118 | IEDLSSQNK |
|  |  | 1016-1024 | SEYESKLSK |
|  |  | 222-229 | NEQYLSYR |
|  |  | 591-599 | SEKMDLESR |
|  |  | 135-143 | KETQSNQQR |
|  |  | 45-57 | HLNDKLLQRNELK |
|  |  | 728-741 | THETLNEYVSCKSK |
|  |  | 1509-1522 | WESEHEQEVSQKIR |
|  |  | 695-711 | LLSNTLDLTKAENDQLR |
|  |  | 1133-1151 | EVNNSTNGPGLNNILITLR |
|  |  | 1375-1394 | IEELQNAKVAQGNNQLEAIR |
|  |  | 302-321 | LVDLLESQLNAVKEELNSIR |
|  |  |  |  |
|  | Mlp2 | 790-797 | LRSDLQSK |
|  |  | 974-981 | NEVERIQK |
|  |  | 843-852 | LSSEIENLDK |
|  |  | 190-200 | LIEEKLSSFSK |
|  |  | 814-825 | WAQNTIDDTEMK |
|  |  | 670-681 | ERMLEEAIDHLK |
|  |  | 1339-1351 | LKAHELQSEDVSR |
|  |  | 1486-1499 | LKENAGSLTFLDNK |
|  |  | 1000-1012 | EMSQYQSTMKENK |
|  |  | 1516-1531 | GNSERPSAVAGFINKQK |
|  |  | 174-188 | DTYRTLMEEIESLK |
|  |  | 863-879 | YDTSVQEKELMLQSK |
|  |  | 104-119 | FLDQNSDASTLEPTLRK |
|  |  |  | QLHVSHEAMREVNDEK |
|  |  |  |  |
|  | Nup60 | 226-235 | TTVYRYSAAK |
|  |  | 464-476 | SSSKGFVFNSVQK |
|  |  | 149-161 | SSKSMITSEGEQK |
|  |  | 448-463 | GDSTPVQPDLSVTPQK |
|  |  | 176-193 | ESGSTPISISNAPTFNPK |
|  |  | 288-307 | NNAASELANPYSSYVSQIRK |
|  |  | 287-306 | KNNAASELANPYSSYVSQIR |
|  |  |  |  |
|  | Mft1 | 01-12 | MPLSQKQIDQVR |
|  |  | 13-26 | TKVHYSEVDTPFNK |
|  |  | 15-33 | VHZSEVDTPFNKYLDILGK |
|  |  | 37-52 | LTGSIINGTLSNDDSK |
|  |  | 201-217 | ELESINSSMKSDIENVR |
|  |  | 218-226 | QEVSSYKEK |
|  |  |  |  |
| *CAF40-TT not3Δ* | Mft1 | 07-14 | QIDQVRTK |
|  |  | 15-33 | VHYSEVDTPFNKYLDILGK |
|  |  | 53-65 | IEKLTEQNISQLK |
|  |  | 100-106 | LENLKDK |
|  |  | 374-392 | KLGGTTSDFSASSSVEEVK |
|  |  |  |  |
|  | Sam1 | 49-61 | TGMIMVFGEITTK |
|  |  | 187-206 | IDTVVVSAQHADEITTEDLR |
|  |  | 237-251 | FVIGGPQGDAGLTGR |
|  |  | 237-252 | FVIGGPQGDAGLTGRK |
|  |  | 301-327 | VQVQFSYAIGIAEPLSLHVDTYGTATK |
|  |  |  |  |
| *CAF130-TT* | Mlp1 | 63-71 | VTVSFDELK |
|  |  | 83-94 | TEMENVIRENDK |
|  |  | 127-135 | LDDLTEEKK |
|  |  | 135-143 | KETQSNQQR |
|  |  | 302-321 | LVDLLESQLNAVKEELNSIR |
|  |  | 336-351 | QTPENEDLLKELQLTK |
|  |  | 574-583 | IESETVNEAK |
|  |  | 969-987 | ISLLKEQMFNLNNELDLQK |
|  |  | 974-987 | EQMFNLNNELDLQK |
|  |  | 1175-1186 | ISLMDVELQDAR |
|  |  | 1213-1227 | LNQLNLLRESNITLR |
|  |  | 1599-1617 | LQEELKDVPHSSHISDDER |
|  |  | 1622-1629 | AEIESRLR |
|  |  | 1665-1673 | MESQLSETK |
|  |  | 1665-1681 | MESQLSETKQSAESPPK |
|  |  | 1737-1745 | HLQNDNDKR |
|  |  |  |  |
| *NOT1-TT caf40Δ* | Mlp1 | 21-36 | LNAIASFFGCSLEQVK |
|  |  | 336-351 | QTPENEDLLKELQLTK |
|  |  | 463-477 | LVECENDLQTLTKQR |
|  |  | 600-611 | IEELQKELEELK |
|  |  | 653-672 | ESTENMSLLNKEIQDLYDSK |
|  |  | 778-792 | DSLRIMVTQLQTLQK |
|  |  | 854-871 | DYESVITSVDSKQTDIEK |
|  |  | 890-906 | LHTYNVMDETINDDSLR |
|  |  | 890-907 | LHTYNVMDETINDDSLRK |
|  |  | 925-943 | EYKDLYETTSQSLQQTNSK |
|  |  | 928-949 | DLYETTSQSLQQTNSKLDESFK |
|  |  | 1050-1061 | HADVSKTISELR |
|  |  | 1133-1152 | EVNNSTNGPGLNNILITLRR |
|  |  | 1175-1188 | ISLMDVELQDARTK |
|  |  | 1221-1236 | ESNITLRNELENNNNK |
|  |  | 1249-1262 | QNVAPIESELTALK |
|  |  | 1563-1577 | IKSMEQSGEIDVVLR |
|  |  | 1665-1681 | MESQLSETKQSAESPPK |
|  |  | 1682-1696 | SVNNVQNPLLGLPRK |
|  |  |  |  |
| *NOT2-TT* | Mlp1 | 144-151 | TLKILDER |
|  |  | 1313-1320 | EELENKER |
|  |  | 1228-1236 | NELENNNNK |
|  |  | 106-115 | FESVENEKMK |
|  |  | 1263-1273 | YSMQEKEQELK |
|  |  | 914-927 | INLTDAYSQIKEYK |
|  |  | 1563-1577 | IKSMEQSGEIDVVLR |
|  |  | 280-299 | GLSDSLNSEKQEFSAEMSLK |
|  |  | 1599-1617 | LQEELKDVPHSSHISDDER |
|  |  | 1375-1394 | IEELQNAKVAQGNNQLEAIR |
|  |  | 1441-1465 | QIQQQLQATSANEQNDLSNIVESMK |
|  |  |  |  |
|  | Mft1 | 07-14 | QIDQVRTK |
|  |  | 15-33 | VHYSEVDTPFNKYLDILGK |
|  |  | 53-65 | IEKLTEQNISQLK |
|  |  | 56-65 | LTEQNISQLLK |
|  |  | 100-106 | LENLKDK |
|  |  | 374-392 | KLGGTTSDFSASSSVEEVK |
|  |  |  |  |
|  | Sam1 | 49 - 61 | TGMIMVFGEITTK |
|  |  | 187 - 206 | IDTVVVSAQHADEITTEDLR |
|  |  | 211 - 220 | SEIIEKVIPR |
|  |  | 229 - 236 | YFIQPSGR |
|  |  | 237 - 251 | FVIGGPQGDAGLTGR |
|  |  | 237 - 252 | FVIGGPQGDAGLTGRK |
|  |  |  |  |
| *NOT2-TT caf40Δ* | Mft1 | 15-33 | VHYSEVDTPFNKYLDILGK |
|  |  | 53-71 | IEKLTEQNISQLKESAHLR |
|  |  | 198-217 | GPKELESINASMKSDIENVR |
|  |  | 201-217 | ELESINASMKSDIENVR |
|  |  | 211-226 | SDIENVRQEVSSYKEK |
|  |  | 249-275 | DGLLNEAEGDNIDEDYESDEDEERKER |
|  |  | 374-392 | KLGGTTSDFSASSSVEEVK |
|  |  |  |  |
| *NOT2-TT*  *caf130Δ* | Dbp5 | 381-392 | SKVLITTNVLAR |
|  |  | 383-392 | VLITTNVLAR |
|  |  |  |  |
|  | Nup2 | 95-101 | ALNLQFK |
|  |  | 239-260 | KTETNAKPFSFSSATSTTEQTK |
|  |  | 48-62 | MAFKPFGSAKSDETK |
|  |  |  |  |
|  | Yra1 | 135-148 | AVERFNGSPIDGGR |
|  |  | 96-107 | EFFASQVGGVQR |
|  |  | 153-165 | LNLIVDPNQRPVK |
|  |  | 8-22 | SLDEIIGSNKAGSNR |
|  |  |  |  |
| *NOT3-TT caf40Δ* | Mft1 | 13-26 | TKVHYSEVDTPFNK |
|  |  | 15-33 | VHYSEVDTPFNKYLDILGK |
|  |  | 201-217 | ELESINASMKSDIENVR |
|  |  | 279-294 | QRSMVEVNTIENVDEK |
|  |  | 374-392 | KLGGTTSDFSASSSVEEVK |
|  |  |  |  |
| *NOT4-TT* | Mlp2 | 982-988 | MIEKIEK |
|  |  | 790-797 | LRSDLQSK |
|  |  | 592-600 | LLASTEENK |
|  |  | 828-836 | SLLTELSNK |
|  |  | 974-981 | NEVERIQK |
|  |  | 843-852 | LSSEIENLDK |
|  |  | 554-563 | VENQTIKEAK |
|  |  | 880-889 | ELEQIQVQLK |
|  |  | 190-200 | LIEEKLSSFSK |
|  |  | 1272-1285 | LSSAENANADLENK |
|  |  | 670-681 | ERMLEEAIDHLK |
|  |  | 1339-1351 | LKAHELQSEDVSR |
|  |  | 1000-1012 | EMSQYQSTMKENK |
|  |  | 1356-1369 | DTYRTLMEEIESLK |
|  |  | 1356-1369 | DTYRTLMEEIESLK |
|  |  | 174-188 | YDTSVQEKELMLQSK |
|  |  | 863-879 | FLDQNSDASTLEPTLRK |
|  |  |  |  |
|  | Sam1 | 2 - 19 | AGTFLFTSESVGEGHPDK |
|  |  | 49 - 61 | TGMIMVFGEITTK |
|  |  | 49 - 61 | TGMIMVFGEITTK |
|  |  | 187 - 206 | IDTVVVSAQHADEITTEDLR |
|  |  | 229 - 236 | YFIQPSGR |
|  |  | 237 - 251 | FVIGGPQGDAGLTGR |
|  |  | 237 - 252 | .FVIGGPQGDAGLTGRK |
|  |  | 301 - 327 | VQVQFSYAIGIAEPLSLHVDTYGTATK |
|  |  | 2 - 19 | AGTFLFTSESVGEGHPDK |
|  |  |  |  |
|  | Sam2 | 51 - 63 | TGMIMVFGEITTK |
|  |  | 51 - 63 | TGMIMVFGEITTK |
|  |  | 231 - 238 | YFIQPSGR |
|  |  | 239 - 253 | FVIGGPQGDAGLTGR |
|  |  | 239 - 254 | FVIGGPQGDAGLTGRK |
|  |  | 303 - 329 | VQVQFSYAIGIAEPLSLHVDTYGTATK |
|  |  |  |  |
| *NOT5-TT*  *caf130Δ* | Mlp2 | 564-575 | DAIIELENINAK |
|  |  | 863-879 | FLDQNSDASTLEPTLRK |
|  |  | 592-613 | LLASTEENKANTNSVTSMEAAR |
